# Chlamydia screening practices among physicians and community nurses in Yukon, Canada

**DOI:** 10.3402/ijch.v72i0.21607

**Published:** 2013-08-05

**Authors:** Karolina Machalek, Brendan E. Hanley, Joy N. Kajiwara, Paula E. Pasquali, Cathy J. Stannard

**Affiliations:** 1 Canadian Public Health Service, Public Health Agency of Canada, Ottawa, Ontario, Canada; 2 Departmment of Health and Social Services, Government of Yukon, Whitehorse, Yukon Territory, Canada; 3 Government of Yukon, Whitehorse, Yukon Territory, Canada

**Keywords:** chlamydia, Yukon Territory, Canada, sexually transmitted diseases, screening, physicians, general practitioners, public health nursing, adolescent

## Abstract

**Background:**

Yukon, a territory in northern Canada, has one of the highest reported sexually transmitted chlamydia infection rates in the country.

**Objective:**

We examined screening practices among physicians and community nurses to elucidate factors that may be contributing to the high rates.

**Design:**

Cross-sectional survey.

**Methods:**

A questionnaire was distributed to all physicians in Yukon and all community nurses in Yukon's communities. We surveyed sexual health assessment frequency, chlamydia testing frequency and barriers to screening. Comparison of physician testing practices was performed to another Canadian jurisdiction, which previously undertook a similar survey. Survey results were compared to the available laboratory data in Yukon.

**Results:**

Eligible physicians and nurses, 79% and 77%, respectively, participated in the survey. Physicians tested 15 to 24-year-old females more frequently than 15 to 24-year-old males for chlamydia (p=0.007). Physicians who asked sexual health assessment questions were more likely to test for chlamydia in both females (p<0.001) and males (p=0.032). More physicians screened females based on risk factors compared to males. General practice physicians in Yukon were more likely to test females for chlamydia than general practice physicians in Toronto, Canada (p<0.001). Community nurses had different testing patterns than physicians, with a lower overall frequency of testing, equal frequency of testing males and females, and in applying risk factor-based screening to both males and females. Barriers to screening included testing causing patient discomfort, patients reluctant to discuss screening, health provider uncomfortable conducting sexually transmitted infection tests and sexual health assessments, among others. Laboratory data in Yukon appear to confirm provider screening patterns.

**Conclusions:**

This survey provides valuable information on health provider screening patterns. We have some evidence which suggests that chlamydia testing rates may be higher among patients seen by physicians in Yukon in comparison to another Canadian jurisdiction. However, more consistent application of optimal screening methods with support to “start the conversation” around sexual health may assist in overcoming barriers to screening and in addressing Yukon's high rate of chlamydia.

Chlamydia, an infection caused by the bacteria *Chlamydia trachomatis*, is the most commonly reported sexually transmitted infection (STI) in Canada (1). Yukon, a territory in northern Canada, has one of the highest chlamydia infection rates in the country. In 2011, chlamydia infection rates were 602.9 per 100,000 population in Yukon, compared to 290.2 per 100,000 population for Canada (2, preliminary data). The epidemiology of chlamydia is similar in Yukon to that of Canada as a whole: rates are highest among females aged 15–24 years and males aged 20–24 years, and reported rates are higher for females than for males (1–3). In Yukon, chlamydia rates are reportedly higher in rural communities than in the urban capital, Whitehorse (3).

Screening^1^ plays an integral role in detecting and treating existing chlamydia cases. Despite the fact that chlamydia can be treated and eradicated, infection is frequently asymptomatic in the early stages among both males and females (4,5). As a result, individuals unaware of their infection status may unknowingly transmit the infection to their sexual partners (4,5). If left untreated, chlamydia can lead to pelvic inflammatory disease (PID) and its sequelae, including chronic pelvic pain, infertility and ectopic pregnancy in women; complications in men include rare cases of epididymoorchitis and infertility (4,5). The economic cost of chlamydia in Canada has recently been estimated to total more than $50 million per year (6).

*Canadian Guidelines on Sexually Transmitted Infections* (STIs) recommend that sexually active females below 25 years of age be routinely screened for chlamydia infection, and the guidelines also recommend it prudent to screen for chlamydia in all sexually active males less than 25 years of age (4). The *Canadian Guidelines on STIs* also specify that screening should be done in accordance with risk factors for chlamydia infection (which include, among others, sexual contact with person(s) with known STI, sexually active youth below 25 years of age, a new sexual partner or more than 2 sexual partners in the past year), identified through STI risk assessments during patient visits (4). The Centers for Disease Control and Prevention in the United States recommend chlamydia screening of all sexually active women aged less than 25 years, and Australian guidelines recommend annual chlamydia testing of all sexually active individuals aged 15–29 years (7,8). However, research has indicated that health providers do not routinely test youth (typically, those aged 15–24 years) for chlamydia in accordance with recommended screening guidelines (9–13). In addition, research has indicated that sexual health risk assessments, which are precursors to chlamydia testing, are not carried out to the extent warranted by screening guidelines (11,14,15).

To our knowledge, no study has established the practice patterns of primary health care providers in a northern Canadian jurisdiction as they relate to the frequency of conducting STI risk assessments and testing for chlamydia. We undertook the current cross-sectional study to contribute to understanding health providers’ STI screening practices in Yukon and to elucidate barriers to discussing and offering screening. We surveyed chlamydia and STI screening practices among health care providers (physicians and community nurses) in Yukon to identify the extent to which screening practices are optimal and reflect Canadian screening guidelines and to elucidate factors that may be contributing to the high rates of chlamydia in Yukon.

## Methods

### Setting

Yukon is one of Canada's 3 northern territories and the westernmost one. It borders Alaska to the west, Beaufort Sea to the north, Northwest Territories to the east and British Columbia (province of Canada) to the south. Yukon has a population of approximately 35,800 people, with 482,443 km^2^ of land (16,17). The capital city is Whitehorse, with a population of approximately 27,000 people (about three quarters of the population of the whole territory). There are 16 main communities, with populations ranging from less than 50 to approximately 2,000 people (17).

Family physicians or general practitioners deliver primary health care in Whitehorse and 2 of the larger Yukon communities, whereas community nurses deliver primary health care in all of Yukon's rural communities. Physicians and most community nurses (those working in rural communities without a physician presence) have in their scope of practice testing for STIs.

### Survey instrument and study group

The survey instrument was adapted with permission from a similar questionnaire carried out by Toronto Public Health in Toronto, Ontario, Canada. The Yukon questionnaire surveyed health providers on the frequency of patients presenting with a sexual health concern or complaint, sexual health assessment (STI risk assessment) frequency and chlamydia testing frequency. Frequencies were surveyed from all types of patient visits in the previous month and were categorized as “never,” “<25%,” “25–50%,” “51–75%” and “76–100%.” Health providers were surveyed separately for male and female patients and by the following age groups: 15–24 years; 25–34 years; 35–50 years; 51–65 years and >65 years. The questionnaire also surveyed barriers to discussing and offering screening; these were close-ended questions. In this report, we have focused on health provider reports specific to the 15 to 24-year-old age group of male and female patients, as this group comprises the majority of chlamydia cases in Yukon.

We distributed the *Physician Survey on STI Screening Practices* to all physicians in Yukon at a Yukon Medical Association meeting in the fall of 2009. The survey consisted of a total of 18 questions: 12 questions on STI screening practices and 6 questions on physician demographic information. The questionnaire was distributed to physicians in a hard copy format for self-completion during this 2-day meeting. Surveys were completed anonymously. The questionnaire was distributed and collected by B.E.H., second author. The majority of physicians completed the survey on site, with a few submitting them to B.E.H. over the next few days.

We adapted the *Physician Survey on STI Screening Practices* to the *Community Nursing Survey on STI Screening Practices* and distributed the survey to community nurses working in Yukon's rural communities in March of 2010 by email, as an attachment to the email in Microsoft Word Document format. The survey consisted of a total of 20 questions: 12 questions on STI screening practices, 6 questions on nurse demographic information and an additional 2 qualitative questions. Surveys were completed anonymously and returned to K.M., first author, by fax, mail or email (using a clinic email address, not a personal email address).

Readers can request a copy of both survey instruments by contacting the corresponding author.

An ethics review was not undertaken since the research was conducted to inform practice and the subjects of the research were health care providers and not patients.

### Analysis

All analyses were conducted using the statistical software SAS version 9.2 (18). To allow for statistical power to make comparisons, most analyses for frequencies of patients presenting with a sexual health concern or complaint, health providers asking sexual health assessment questions and health providers testing for chlamydia were sub-categorized into 2 groups: the first category was “less than or equal to 50%, or half, of the time,” which included the categories “never,” “<25%,” and “25–50%,” and the second category was “greater than 50%, or half, of the time,” which included the categories “51–75%” and “76–100%.” Chi-square analysis was used to test for differences in proportions, except when the count in a cell was below 5, when the Fisher's exact test was used. Inferential statistical analyses for data from the *Community Nursing Survey on STI Screening Practices* were not performed due to the small sample size (n=20) and the low variability of data among respondents.

With permission from the lead author and Toronto Public Health, we compared physician sexual health assessment frequencies and physician chlamydia testing frequencies from the Yukon *Physician Survey on STI Screening Practices* to published results from Toronto Public Health which undertook a similar survey in Toronto, Ontario, Canada (9). We compared Yukon physician data for 15 to 24-year-old patients to Toronto physician data for 15 to 19 and 20 to 24 year-old patients, since the Yukon survey categorized these age groups as one. We restricted this analysis to general practice physicians who answered the Yukon survey, since the Toronto survey was limited to general practice physicians.

We compared the survey results of physician-reported chlamydia testing frequency to the number of chlamydia laboratory tests ordered in Yukon per year, from 2007 to 2011. We compared the proportion of laboratory tests done for females to those done for males.

## Results

### Study participant characteristics

A total of 42 physicians participated in the survey of 53 eligible, with a response rate of 79%.^2^ The majority (86%) of physicians practiced only in Whitehorse (which is representative of the territory as a whole). Almost all (95%) of the physicians surveyed were general practice physicians and 52% were female.

Twenty community nurses participated out of 26 eligible, with a 77% response rate. The majority (80%) of nurses were female and all practiced in Yukon's rural communities.

### How frequently is sexual health a part of the conversation?

**Fig. 1:**
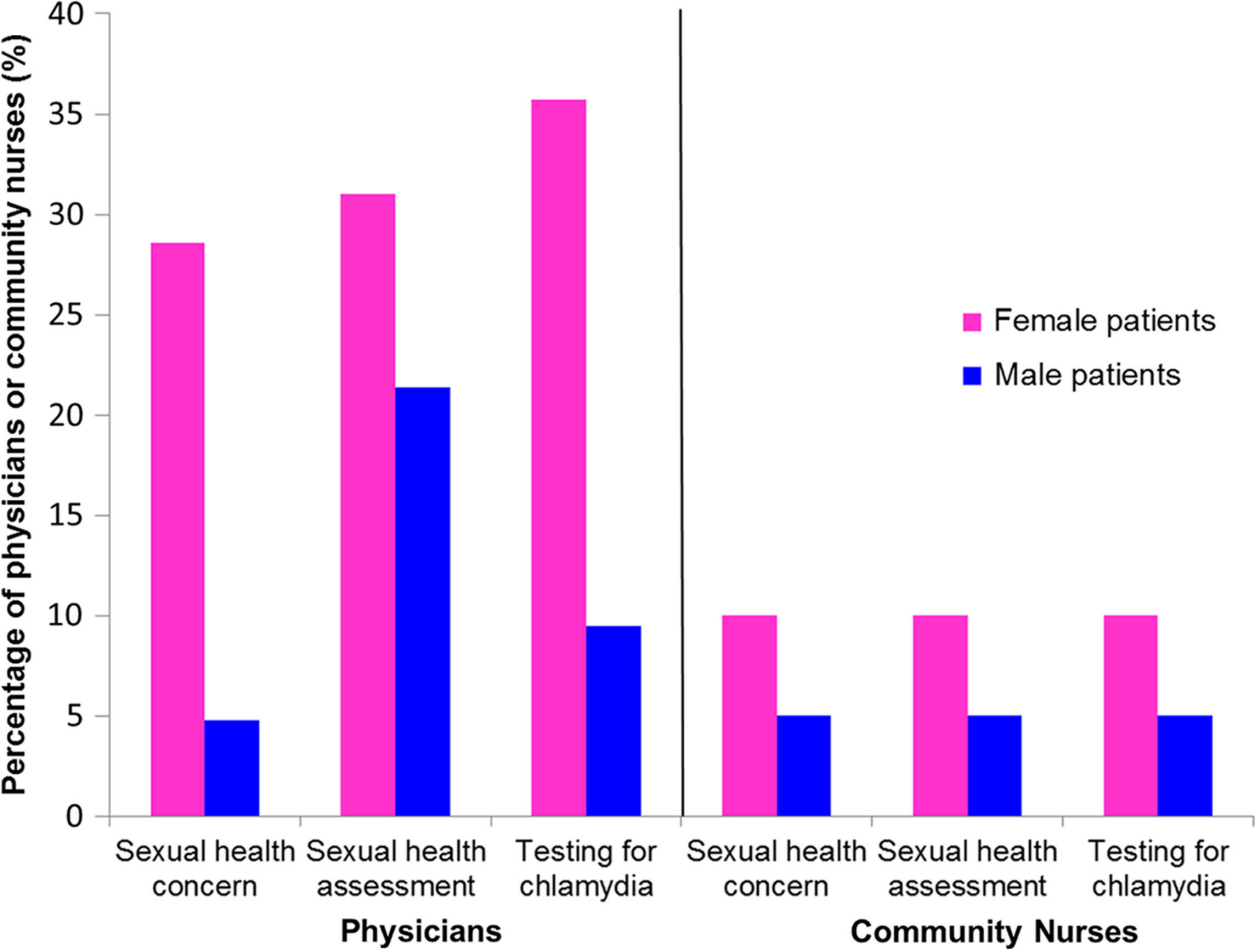
The percentage of physicians (n = 42, 2009 data) and community nurses (n = 20, 2010 data) in Yukon, Canada reporting 15–24 year-old female and male patients presenting with a sexual health concern or complaint, conducting sexual health assessments, and testing for chlamydia greater than half the time as a proportion of all visits in their practice in the previous month.

Nearly a third (29%) of physicians reported 15 to 24-year-old female patients presenting with a sexual health concern more than half the time in the previous month, compared to just 5% of physicians who said the same for 15 to 24-year-old male patients (p=0.004) (Fig. 1). Similarly, 31% of physicians reported conducting sexual health risk assessments on 15 to 24-year-old female patients at least half the time in the previous month, compared to 21% of physicians who reported the same for males (p=0.463) (Fig. 1). Just over a third (36%) of physicians reported testing 15 to 24-year-old females for chlamydia at least half the time in the past month, compared to 10% of physicians who tested 15 to 24-year-old males for chlamydia (p=0.007) (Fig. 1).

Nearly one-fifth (19%) of physicians routinely (76–100% of the time) asked 15 to 24-year-old female patients sexual health assessment questions. Only 1 physician (2%) routinely asked 15 to 24-year-old male patients sexual health assessment questions.

Unlike physicians, community nurses did not display differences in frequencies of patients coming in with a sexual health concern, conducting STI risk assessments and testing for chlamydia. Just 10% of nurses reported their female patients coming in with a sexual health concern or complaint at least half the time in the past month, conducting sexual health risk assessments at least half the time in the past month, and testing for chlamydia at least half the time in the past month among their female patients; 5% of nurses reported the same for males (Fig. 1). Only 1 nurse (5%) routinely (76–100% of the time) conducted STI risk assessments.

Community nurses conducted STI risk assessments and tested for chlamydia less frequently in both their female and male patients than physicians (Fig. 1). Just 10% of community nurses reported conducting sexual health assessments at least half the time among their 15 to 24-year-old female patients compared to 31% of physicians who did the same (Fig. 1). Among males, just 5% of community nurses conducted STI risk assessments more than half the time compared to 21% of physicians who did so (Fig. 1). With regard to testing for chlamydia at least half the time in the past month, 10% of community nurses did so among their 15 to 24-year-old female patients, compared to 36% of physicians who reported the same (Fig. 1). Among males, just 5% of community nurses reported testing for chlamydia more than half the time, compared to 10% of physicians who reported the same (Fig. 1).

### Factors associated with testing for chlamydia

We found that physicians who tested patients for chlamydia were more likely to have patients see them with a sexual health concern or complaint and to have asked their patients about sexual health (Table I). It is interesting to note that the odds of physicians testing patients who came in with a sexual health concern or complaint were more pronounced for male patients than for female patients (Table I). At the same time, the odds of physicians testing patients whom they asked about sexual health were similar for male patients and female patients (Table I).

**Table I:** The association of patients presenting with a sexual health concern or physicians asking sexual health assessment questions and physicians testing for chlamydia (2009 data, Yukon, Canada)

	Tested for chlamydia					
	
	15 to 24-year-old female patients			15 to 24-year-old male patients		
		
	Yes^a^	No^a^		Yes^a^	No^a^	
				
Context of patient visit	N (%*) of physicians (n=42)		OR (95% CI)	N (%*) of physicians (n=39)		OR (95% CI)
Patient presented with a sexual health concern						
Yes^b^	8 (19.0)	4 (9.5)	6.6 (1.5–28.5) p=0.013	3 (7.7)	3 (7.7)	32.0 (2.5–411.4) p=0.008
No^b^	7 (16.7)	23 (54.8)		1 (2.6)	32 (82.0)	
Physician asked sexual health assessment questions						
Yes^c^	10 (23.8)	3 (7.1)	16.0	3 (7.7)	6 (15.4)	14.5 (1.3–164.4) p=0.032
No^c^	5 (11.9)	24 (57.1)	(3.2–80.1) p<0.001	1 (2.6)	29 (74.4)	

* Based on the number of physicians who answered each question; may not add up to 100% due to rounding.

OR=odds ratio; CI=confidence interval.

a “Yes” represents testing for chlamydia >50% of the time and “No” represents testing for chlamydia ≤50% of the time.

b “Yes” represents patients presenting with a sexual health concern >50% of the time for female patients and ≥25% of the time for male patients; “No” represents patients presenting with a sexual health concern ≤50% of the time for female patients and <25% of the time for male patients (male cut-offs were lower than female cut-offs due to inadequate sample sizes for males in the category “>50% of the time”).

c “Yes” represents physicians asking sexual health assessment questions >50% of the time and “No” represents physicians asking sexual health assessment questions ≤50% of the time.

We found that more physicians reported screening for chlamydia based on risk factors among females than among males (Table II). For example, 86% of physicians reported that “multiple sexual partners” would be a trigger for chlamydia screening (indicator that would prompt or result in laboratory testing) among females, but only 48% reported the same for males. Similarly, 57% of physicians reported screening females based on the trigger of “patient is known to be sexually active” compared to 19% of physicians who reported the same for males. We also found that physicians who reported testing their 15 to 24-year-old male patients for chlamydia at least half the time in the past month had increased odds of screening 15 to 24-year-old males based on the trigger of “patient is known to be sexually active” (OR=18.0, 95% CI=1.5–209.3, p=0.022).

**Table II:** Percentage of physicians (n=42, 2009 data) and community nurses (n=20, 2010 data) in Yukon, Canada, reporting triggers for screening (indicators for laboratory testing) for chlamydia among their female and male patients

	Physicians Number (%)^a^		Community nurses* Number (%)^a^	
		
Trigger for screening	Female patients	Male patients	Female patients	Male patients
Patient asks to be screened for chlamydia or STIs	42 (100.0)	31 (73.8)	19 (95.0)	19 (95.0)
Patient is symptomatic	41 (97.6)	31 (73.8)	16 (80.0)	17 (85.0)
Patient reports being a contact or is named a contact of a positive *Chlamydia trachomatis* case	38 (90.5)	30 (71.4)	18 (90.0)	18 (90.0)
Patient has multiple sexual partners	36 (85.7)	20 (47.6)	15 (75.0)	13 (65.0)
Patient is young adult (aged 17–25 years)	28 (66.7)	10 (23.8)	10 (50.0)	10 (50.0)
Patient is known to be sexually active	24 (57.1)	8 (19.1)	14 (70.0)	12 (60.0)
Patient is being seen for birth control	25 (59.5)	2 (4.8)	15 (75.0)	13 (65.0)
Screening is a standard addition to Pap testing	27 (64.3)	N/A	18 (90.0)	N/A
Screening is a standard addition to annual check-ups	16 (38.1)	1 (2.4)	14 (70.0)	10 (50.0)

* 1 nurse (5.0%) noted that this question was out of the scope of practice.

a Percentages are reported to 1 decimal place in the Table, whereas they are rounded to the nearest whole number in the text.

In contrast to physicians, we found that equally as many nurses screened based on risk factors among females and among males (Table II).

Notably, the majority of physicians (64%) and the vast majority of community nurses (90%) reported that screening is a standard addition to Pap testing among females. In total, 38% of physicians and 70% of community nurses reported that screening is a standard addition to annual check-ups for females. Only 2% of physicians reported that screening is a standard addition to annual check-ups for males, whereas 50% of community nurses reported the same for their male patients.

### Comparisons with other jurisdiction

While Yukon and Toronto general practice physicians reported similar frequencies of asking about sexual health among their 15 to 24-year-old female patients, Yukon general practice physicians reported that they test for chlamydia more frequently than Toronto general practice physicians (Table III). Yukon general practice physicians had 3.7–5.6 times the odds of testing their 15 to 24-year-old female patients for chlamydia than Toronto general practice physicians (Table IV).

**Table III:** Comparison of self-reported frequencies for general practice physicians from Yukon, Canada (2009 data), and general practice physicians from Toronto, Ontario, Canada (2006 data), asking sexual health assessment questions and testing for chlamydia among their 15 to 24-year-old female patients

	Frequency as a proportion of female patients in the previous month (%)	Yukon general practice physicians (for 15 to 24-year-old female patients) N (%)	Toronto general practice physicians (for 15 to 19-year-old female patients) N (%)*	Toronto general practice physicians (for 20 to 24-year-old female patients) N (%)*	Chi-square
Asked sexual health assessment questions	<25	14 (35.0)	102 (41.5)	87 (35.4)	
	25–50	13 (32.5)	50 (20.3)	59 (25.9)	
	51–75	5 (12.5)	40 (16.3)	40 (16.3)	
	76–100	8 (20.0)	54 (22.0)	60 (24.4)	
	Total	40	246	246	4.565 (p=0.601)
Tested for chlamydia	<25	17 (42.5)	166 (68.3)	150 (62.7)	
	25–50	8 (20.0)	51 (21.0)	59 (23.9)	
	51–75	6 (15.0)	14 (5.8)	20 (8.1)	
	76–100	9 (22.5)	12 (4.9)	18 (7.3)	
	Total	40	243	247	22.998 (p<0.001)

* Numbers taken from Hardwick, McKay and Ashem (9) and published with permission from lead author and Toronto Public Health.

**Table IV:** Association of physician practice location and physician-reported frequency of testing for chlamydia as a proportion of all patient visits in the previous month (2009 data for Yukon, Canada; 2006 data for Toronto, Ontario, Canada)

	Number of general practice physicians reporting frequency of testing for chlamydia as a proportion of all visits in their practice in the previous month among 15 to 24-year-old patients in Yukon and 15 to 19-year-old patients in Toronto*		
		
Physician practice location	>75% of the time	≤75% of the time	OR (95% CI)
Yukon (15–24)	9	31	5.6 (2.2–14.3) p<0.001
Toronto (15–19)*	12	231	
	Number of general practice physicians reporting frequency of testing for chlamydia as a proportion of all visits in their practice in the previous month among 15–24 year-old patients in Yukon and 20–24 year-old patients in Toronto*		
		
Physician practice location	>75% of the time	≤75% of the time	OR (95% CI)

Yukon (15–24)	9	31	3.7 (1.5–8.9) p=0.006
Toronto (20–24)*	18	229	

* Numbers taken from Hardwick, McKay and Ashem (9) and published with permission from lead author and Toronto Public Health.

OR=odds ratio; CI=confidence interval.

### Barriers to screening

**Fig. 2:**
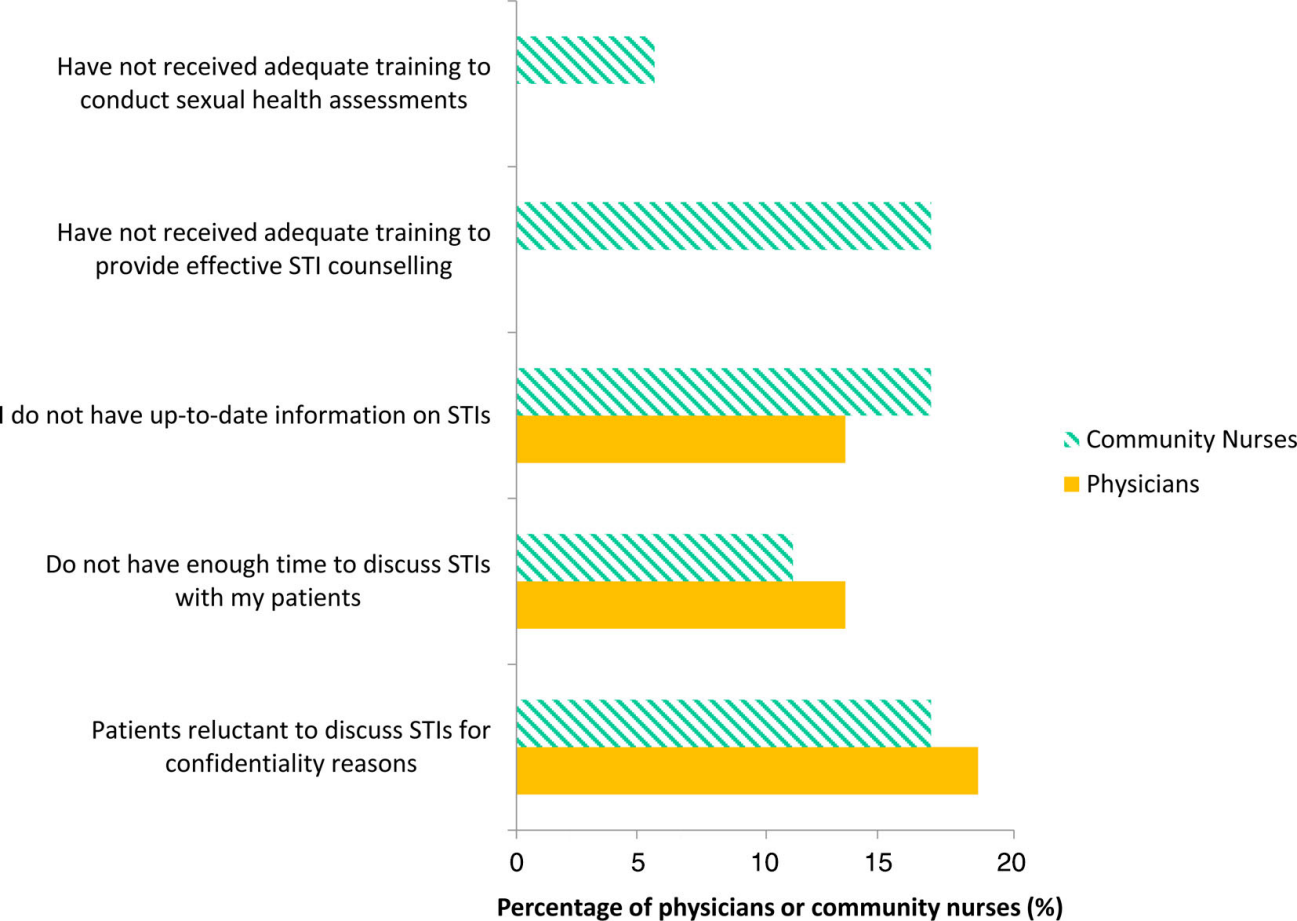
Percentage of physicians (n=42, 2009 data) and community nurses (n=20, 2010 data) in Yukon, Canada reporting barriers to discussing chlamydia screening with their patients.

The most commonly reported barriers to discussing screening for physicians and community nurses were: patients reluctant to discuss STIs for reasons of confidentiality (not defined in the survey, but commonly understood to mean privacy, particularly in reference to residing in small communities), physicians/nurses not having enough time to discuss STIs with patients and physicians/nurses not having up-to-date information on STIs. A minority of community nurses also reported not having adequate training to conduct sexual health assessments and STI counselling (Fig. 2).

**Fig. 3:**
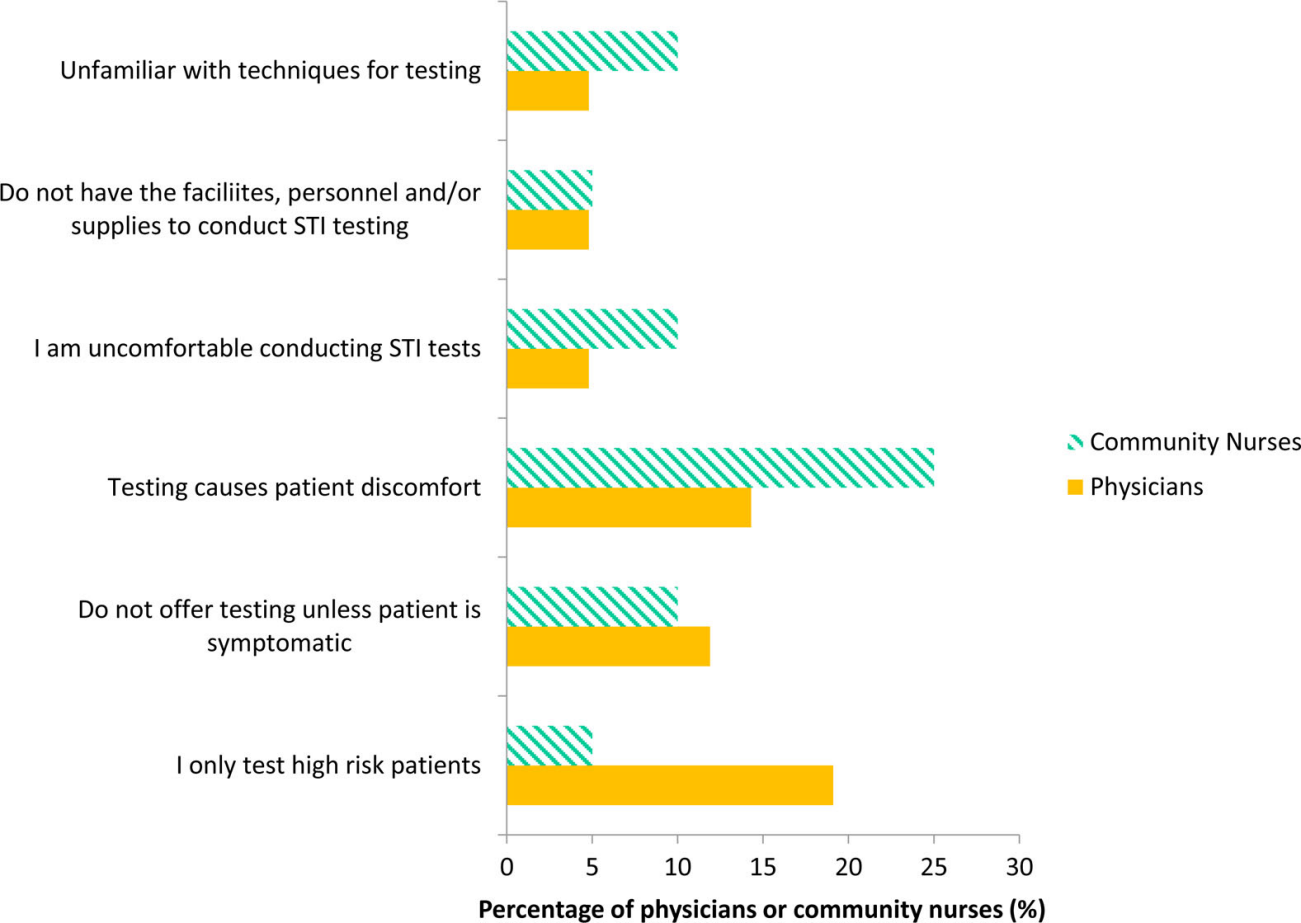
Percentage of physicians (n=42, 2009 data) and community nurses (n=20, 2010 data) in Yukon, Canada reporting barriers to offering chlamydia screening to their patients.

With regard to barriers to offering screening, 25% of community nurses identified “testing causes patient discomfort” as a barrier to screening (this was prior to urine testing being introduced and widely available). Nearly 20% of physicians reported that they only test high-risk patients. A minority of physicians and community nurses indicated that they do not offer testing unless the patient is symptomatic, they are uncomfortable with conducting STI tests, unfamiliar with techniques for testing and do not have the facilities or personnel to conduct testing (Fig. 3).

### Laboratory data

**Fig. 4:**
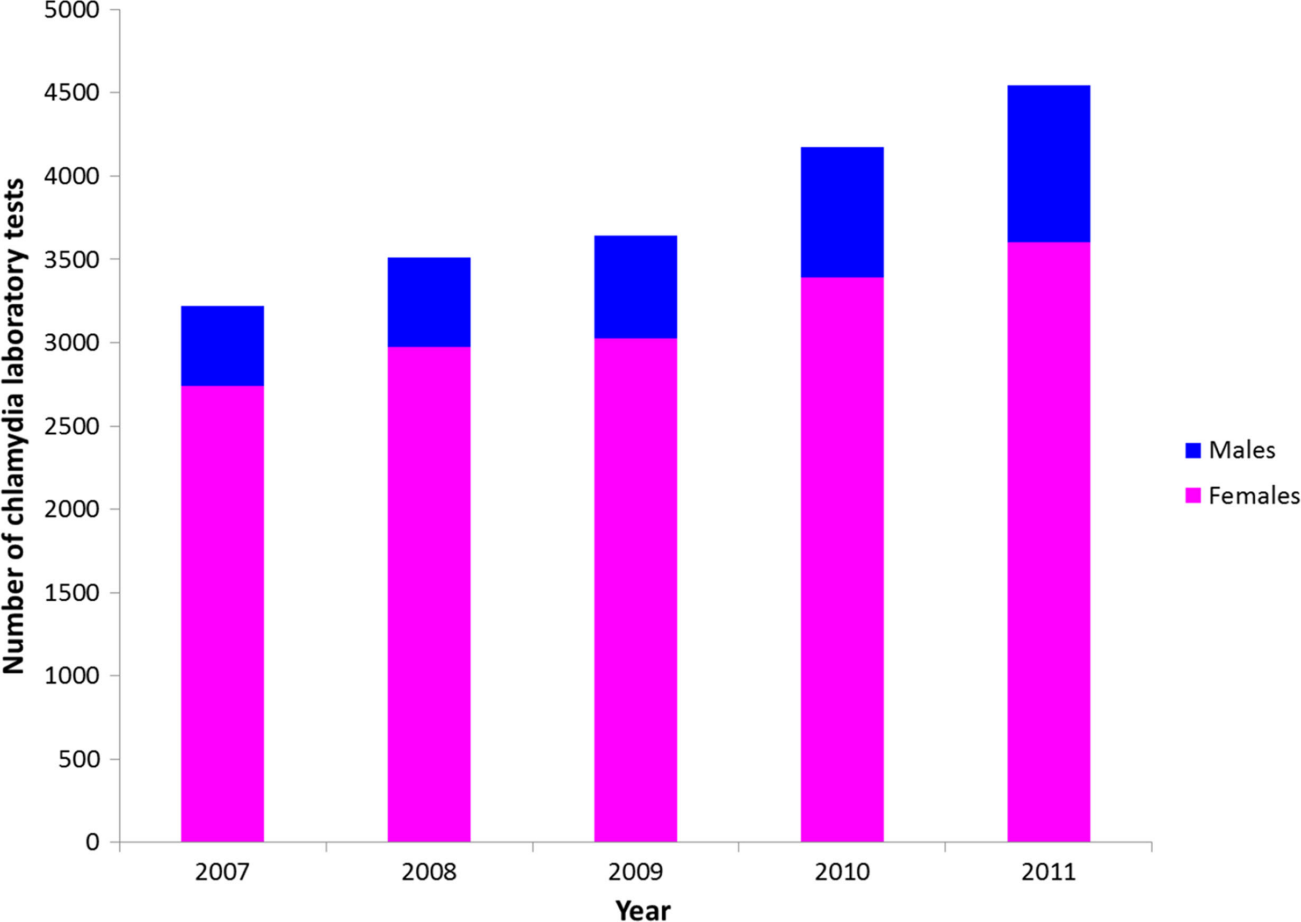
Number of chlamydia laboratory tests conducted in Yukon, Canada for females and for males, 2007–11. Numbers of lab tests by sex were imputed for 2010, calculated based on the total number of chlamydia lab tests for 2010 and an average proportion of tests for males and females between 2009 and 2011.

According to laboratory data, the vast majority of chlamydia lab tests in Yukon are conducted among females (average of 83% of all tests between 2007 and 2011) (Fig. 4). Between 2007 and 2011, the number of tests conducted among females rose by 31% and the number of tests conducted among males rose by 97%.

## Discussion

To our knowledge, this study is the first to document health provider STI screening practices in a northern Canadian jurisdiction. We found a difference among physicians, but not community nurses, in how sexual health is addressed among female and male patients (Fig. 1). Sexual health concerns are a more frequent reason for visits at physician offices among female patients than male patients. The frequency that sexual health assessment questions are being asked by physicians has relatively little difference between females and males compared to how frequently physicians are testing – testing more frequently among females than among males. In contrast, community nurses reported similar frequencies of female and male patients coming in with a sexual health concern, asking sexual health assessment questions and testing for chlamydia. Laboratory data corroborate the findings from the physician survey, since, on average, 83% of chlamydia lab tests are done for females (Fig. 4). This may point to a degree of routine chlamydia screening during Pap testing or annual check-ups among females, since 64% of physicians reported screening as a standard addition to Pap testing and 38% reported screening as a standard addition to annual check-ups among females (Table II); screening for chlamydia as a standard addition to Pap testing and during annual check-ups has been observed among clinicians elsewhere (9). Interestingly, more physicians reported conducting sexual health assessments than testing for chlamydia among male patients (Fig. 1). This may highlight the lack of routine screening among males, in contrast to females, as only 2% of physicians reported that screening is a standard addition to annual check-ups for their male patients (Table II). For those physicians who asked males sexual health assessment questions and did not test males for chlamydia, it may be that physicians identified them as low risk and did not proceed to testing or physicians may have treated them on suspicion of STI rather than proceed to testing; for example, 74% of physicians reported that they would screen male patients if they asked to be screened for chlamydia or if they were symptomatic, compared to 100% and 98% of physicians, respectively, who reported they would test female patients if they asked to be screened or if they were symptomatic (Table II).

Most importantly, what we found is, physicians that tested for chlamydia had a similar rate of conducting STI risk assessments among female and male patients (Table I). In other words, if the question is asked about sexual health, physicians are more likely to test for chlamydia in both females and males – we termed this relationship “Ask and Test.” This underscored the importance of conducting STI risk assessments, as recommended by the *Canadian Guidelines on STIs* (4). We also found that the odds of a sexual health concern triggering testing for chlamydia were higher among males than among females (Table I). This may be due to the fact that females present with a greater variety of sexual health concerns than males (e.g. requesting contraception), whereas for males, the primary reason for coming in with a sexual health concern is likely due to STI. We found that more physicians reported screening for chlamydia based on risk factors among females than among males (Table II). Risk factors may be more apparent with a female patient requesting contraception, since she has implicitly informed her provider that she either is or intends to become sexually active, and may be at risk for STIs. In our survey, 60% of physicians reported “patient being seen for birth control” as a trigger for chlamydia screening among female patients. For male patients, there is no similar trigger that provides information about risk. These distinctions would likely have an effect on the sex differences noted in patients presenting with a sexual health concern, risk assessment and testing frequencies. It may also be the case that physicians put more value on screening females than on screening males due to the greater and more frequent health consequences of undetected and untreated chlamydia in females (PID, chronic pelvic pain, ectopic pregnancy and infertility); however, we did not survey the reasons for these differences. Further research should investigate the reasons for these discrepancies.

Importantly, physicians who did report testing young male patients for chlamydia more than half the time had increased odds of screening males based on the risk factor of being sexually active. This suggests that correctly applying risk factors is associated with testing for chlamydia.

When we compared our survey results to those of a similar survey conducted in Toronto, Ontario, Canada, by Toronto Public Health, we found that general practice physicians in Yukon were equally likely to conduct sexual health risk assessments but more likely to test their 15 to 24-year-old female patients for chlamydia than general practice physicians in Toronto (Tables III and IV). These self-reported results from surveys of physician screening practices may indicate that Yukon physicians likely screen for chlamydia more frequently, at least among 15 to 24-year-old females, than physicians in Toronto, and potentially elsewhere. These data also support the hypothesis that Yukon's chlamydia testing rates (frequency of testing for chlamydia) may be higher than other jurisdictions, which may be leading to increased reported rates of chlamydia, due to the detection of more cases through a higher screening rate. This comparison of screening rates based on survey data has been corroborated by Yukon laboratory data comparisons to other jurisdictions, which we have reported elsewhere (19). Briefly, we compared Yukon laboratory data on testing volume (number of chlamydia laboratory tests performed as a proportion of the population) and positivity (percentage of tests that were positive) to 2 other Canadian jurisdictions with available data: Saskatchewan, Canada and British Columbia Centre for Disease Control (BCCDC), in British Columbia, Canada. Average chlamydia testing volume in Yukon between 2000 and 2011 was 10.9%; average chlamydia positivity in Yukon between 2000 and 2011 was 5.5% (19). Average testing volume in Saskatchewan between 2000 and 2007 was 4.1%, and average positivity in Saskatchewan between 2000 and 2007 was 8.9% (20). Average chlamydia positivity at BCCDC was approximately 6% in 2010 (21). Based on these comparisons of laboratory data, Yukon appears to have conducted a greater number of chlamydia tests as a proportion of the population than Saskatchewan (10.9% in Yukon versus 4.1% in Saskatchewan). At the same time, despite the apparently higher testing rates in Yukon, the percentage of tests that were positive was on par or lower in Yukon than BCCDC and Saskatchewan (5.5% in Yukon versus approximately 6% at BCCDC and 8.9% in Saskatchewan). These laboratory data comparisons, although limited, appear to support the results of the physician survey and suggest that Yukon's chlamydia testing rates may be higher than other jurisdictions. This, in turn, may account for some of the increased reported rates of chlamydia in Yukon due to the detection of more cases through a higher screening rate.

Our research indicates that community nurses have different asking and testing patterns than physicians in Yukon. Community nurses did not report differences between the percentage of patients coming in with a sexual health concern, asking sexual health assessment questions and testing for chlamydia (Fig. 1). These data suggest that in the context of rural Yukon and for community nursing practice, STI risk assessments and chlamydia testing may only be taking place during visits that are specific to a sexual health concern or complaint. As a result, patients who do not present with a complaint but may be at risk for chlamydia may not be identified. The survey data also indicate that chlamydia testing rates are reportedly lower for community nurses than for physicians (Fig. 1). These data may point to a degree of under-screening in rural Yukon, where community nurses predominantly practice, compared to urban Yukon, where physicians predominantly practice, which may suggest that reported chlamydia rates in rural Yukon are actually lower than expected, despite the fact that they are on the whole higher than rates in Whitehorse, the urban capital (3). A recent study from the United States also found a similar discrepancy between testing rates in urban and rural environments, and found that the frequency of self-reported HIV testing decreased substantially as the residential environment became progressively more rural (22).

Data from the community nursing survey on triggers for chlamydia screening show that equally as many nurses screen based on risk factors among females and among males, in contrast to physicians. These data suggest that the majority of nurses know the risk factors for screening, but this knowledge may not be translating into practice. During a knowledge exchange session with the community nurses to try to explain the survey results, barriers to discussing and offering screening were highlighted as a reason for this discrepancy. It was suggested that nurses do not necessarily have the time to conduct sexual health assessments and chlamydia screening when a young patient comes to clinic since their time in clinic is primarily occupied by acute cases (e.g. a broken arm) and there may not be a good or right opportunity for broaching the subject with patients; however, only 10% of community nurses identified lack of time as a barrier to discussing chlamydia screening in the survey (Fig. 2). The knowledge exchange session also identified the difficulty of getting young patients, especially males, to come into clinic and the difficulty in broaching the subject with patients, since 15% of nurses identified patients reluctant to discuss STIs for reasons of confidentiality, possibly due to patients’ perceived lack of privacy as a result of residing in small communities (Fig. 2). There may be other factors that this survey did not identify specific to a rural environment that may be leading to low asking and testing patterns among community nurses. Further research should investigate such factors. Importantly, the differences between the results of the physician and community nursing survey indicate that the context of practice has an effect on opportunities for discussing and offering chlamydia and STI screening, and the need to specifically target interventions designed to increase opportunities for testing in each context and to each type of health care provider.

### Strengths and limitations

Strengths of this study included the high response rate of the study participants, which makes it likely that our study results are generalizable to the study population (primary health care providers in Yukon). However, a limitation of the study is the lack of generalizability to other populations. The small sample size was a limitation in being able to undertake inferential analyses, especially among data specific to community nurses. As the original survey instrument was designed for a large urban population in Toronto, despite it being adapted to the Yukon context, it may not have served to fully illuminate the contextual differences in barriers to assessment and screening in Yukon's unique northern rural communities.

Additionally, there is a potential for bias in self-reported data. Providers may have recalled their practice habits differently than what they actually do, and may have sought to provide the “socially desirable” response. At the same time, however, we do have laboratory data to substantiate reports from the physician survey – the overall percentage of tests done for females and males (Fig. 4) appears to support physician-reported data (Fig. 1). Further dividing the laboratory data by provider type would provide another test for internal validity; unfortunately, this kind of analysis of laboratory data was not available.

This survey also assessed supports either utilized or required (not reported in this article), which has allowed us to take immediate action to improve supports to health care providers in Yukon. A Chlamydia Reduction Strategy Working Group has been formed at the Department of Health and Social Services, Government of Yukon which has utilized the survey results to design health provider education, including making screening guidelines easily available, providing additional training opportunities and reminding physicians and nurses to “ask and test” (e.g. through a quarterly surveillance newsletter sent out to all health care providers in Yukon). This practice-based survey has allowed us to improve professional education in the area of STIs and at the same time contribute to a body of research on the factors that are associated with STI risk assessments and STI testing.

## Conclusions

This survey provides valuable information on health provider screening patterns in Yukon. We found that screening patterns vary between physicians and community nurses, and that there is a relationship between conducting STI risk assessments and testing for chlamydia, as well as the importance of applying risk factor knowledge to practice. We also identified some barriers to discussing and offering screening, which should be addressed and mitigated. We have some evidence which suggests that chlamydia testing rates may be higher among patients seen by physicians in Yukon in comparison to Toronto, Canada. Our laboratory data comparisons with Saskatchewan, Canada (20), and BCCDC, Canada (21), which we have reported elsewhere (19), also suggest that Yukon's chlamydia testing rates may be higher than those in other jurisdictions; however, data are limited.

*Canadian Guidelines on STIs* recommend conducting sexual health risk assessments for sexually active youth less than 25 years of age, which is a known risk factor for STIs. According to our survey results, only about 19% of physicians in Yukon conduct sexual health assessments routinely among females in that age group, and only 2% do so among males. Similarly, only 5% of community nurses routinely conduct sexual health assessments among females and males in that age group. These data point to the need to start the conversation around sexual health for health care providers with their patients, as health providers may be missing important risk factor information that may place patients at risk for STIs. More consistent application of optimal screening methods with support to “start the conversation” around sexual health may assist in overcoming barriers to screening and in addressing Yukon's high rate of chlamydia.

## Footnotes

1 Screening is explicitly defined neither in the *Canadian Guidelines on Sexually Transmitted Infections* (STIs) nor in our survey instrument. Screening could be defined as “case-finding for patients with asymptomatic disease” and the implication is for case-finding by lab testing.

2 Percentages are rounded to the nearest whole number in the text of this paper for readability, whereas they are reported to 1 decimal place in the Tables and Figures for precision.
